# Novel Approaches for BAV Aortopathy Prediction—Is There a Need for Cohort Studies and Biomarkers?

**DOI:** 10.3390/biom8030058

**Published:** 2018-07-19

**Authors:** Evaldas Girdauskas, Johannes Petersen, Niklas Neumann, Shiho Naito, Tatiana Gross, Annika Jagodzinski, Hermann Reichenspurner, Tanja Zeller

**Affiliations:** 1 Department of Cardiovascular Surgery, University Heart Center, Martinistraße 52, 20246 Hamburg, Germany; joh.petersen@uke.de (J.P.); n.neumann@uke.de (N.N.); s.naito@uke.de (S.N.); t.sequeira-gross@uke.de (T.G.); reichenspurner@uke.de (H.R.); 2 German Center for Cardiovascular Research (DZHK), Partner Site Hamburg/Lübeck/Kiel, 20246 Hamburg, Germany; a.jagodzinski@uke.de (A.J.); t.zeller@uke.de (T.Z.); 3 Department of General and Interventional Cardiology, University Heart Center Hamburg, 20246 Hamburg, Germany

**Keywords:** bicuspid aortic valve, aortopathy, biomarkers, microRNAs

## Abstract

Bicuspid aortic valve (BAV) disease is the most common congenital malformation of the human heart with a prevalence of 1–2% in the general population. More than half of patients with a BAV present with a dilated proximal aorta (so-called bicuspid aortopathy) which is associated with an enhanced risk of life-threatening aortic complications. Up to now, the pathogenesis of bicuspid aortopathy as well as the risk stratification of aortic complications has not yet been sufficiently clarified. Recent findings have shown that bicuspid aortopathy features phenotypic heterogeneity. Two distinct valvulo-aortic phenotypes, the so-called root phenotype, as well as a dilation of the tubular ascending aorta, coincide with a significantly different risk for aortal complications. However, the phenotype-based classification that is only based on these two clinical forms is not sufficient to estimate the risk of aortal complications in a prognostically relevant way. Therefore, there is growing clinical interest to assess novel approaches in BAV research and to introduce circulating biomarkers as an elegant diagnostic tool to improve risk stratification in BAV aortopathy. A large scale epidemiological cohort study, ranking from apparently healthy individuals to disease patients, and comprehensive biobanks provide the opportunity to study BAV disease and its complications and to identify novel biomarkers for BAV aortopathy surveillance and prognosis. Firstly, the data indicate that several protein-based biomarkers and non-coding RNA molecules, in particular circulating microRNAs, can serve as relevant molecular biomarkers to predict the course of BAV-associated aortopathy. Here, we review the current literature and knowledge about BAV from a clinical point of view, and report about novel approaches in BAV biomarker research.

## 1. BAV–The Clinical Perspective

### 1.1. Controversy of BAV Aortopathy and Current Clinical Diagnostic Tools

**Figure 1:**
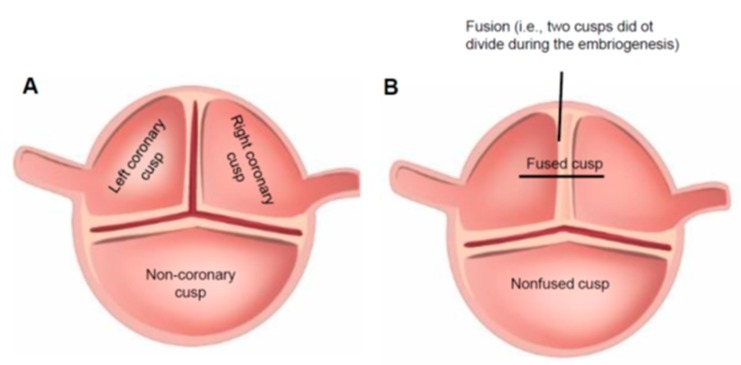
(**A**) Normal tricuspid aortic valve; (**B**) bicuspid aortic valve. There is an area of fusion between two adjacent cusps, which indicates the lack of splitting between the right- and left-coronary cusp.

Bicuspid aortic valve disease (BAV) is the most common anomaly of the human heart [1,2]. In contrast to a “normal” tricuspid aortic valve (TAV) (Figure 1A), a BAV has only two cusps (Figure 1B) that may differ considerably in their size and configuration. BAV development is characterized by the lack of splitting of two adjacent cusps during valvulogenesis, where the degree of the persisting congenital fusion (raphe) is variable [3].

The prevalence of this congenital defect amounts to 1–2% of the general population [1,2]. The mode of inheritance of the bicuspid aortic valve is autosomal-dominant with variable expression and incomplete penetrance [4,5].

**Figure 2:**
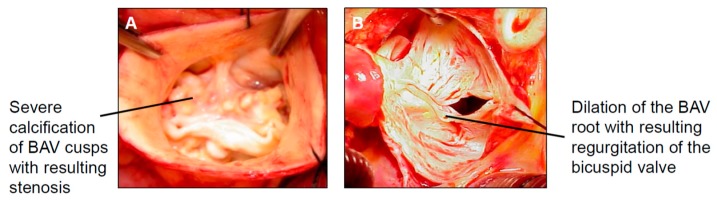
Accelerated BAV degeneration with (**A**) resultant valvular stenosis and/or (**B**) regurgitation.

BAV leads to accelerated degeneration of the valvular structures in the majority of BAV patients, which results in valvular stenosis (Figure 2A) and/or regurgitation (Figure 2B) [6].

Due to the high prevalence of this congenital anomaly in the population and the life-long increased risk of adverse cardiovascular events (i.e., risk of developing aortic stenosis and regurgitation, endocarditis, and aortic dissection/rupture), BAV is a disorder that places a considerable burden on public health [7].

Morbidity in BAV patients is significantly higher than in patients with any other congenital anomaly of the heart [8]. Also illustrating the high relevance of this morbid condition is the patient organization that has been founded in the US (Bicuspid Aortic Foundation, Laguna Niguel, CA, USA), which is dedicated specifically to patients suffering from BAV disease—http://bicuspidfoundation.com. In Germany and in Europe, there is currently no comparable initiative. 

### 1.2. Definition of Aortopathy

**Figure 3:**
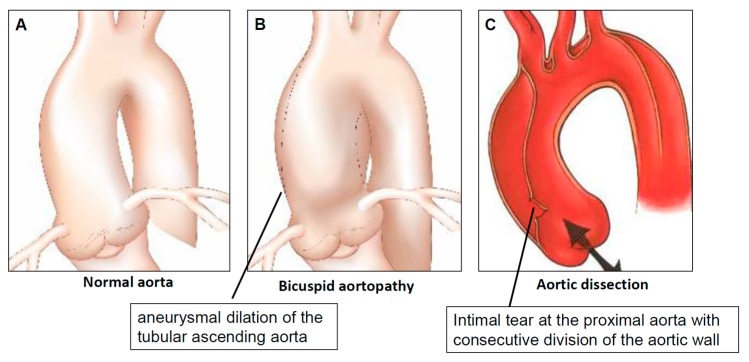
Complications of bicuspid aortopathy. (**A**) Normal aorta; (**B**) aneurysm of the acending aorta; (**C**) acute aortic dissection (type **A**).

Any proximal aortic disease that is associated with a maximum aortic diameter > 40 mm and/or an indexed aortic diameter exceeding 21 mm/m^2^, respectively, is defined as aortopathy [9]. Other authors postulated the benefit of using indexed aortic area (i.e., aortic root or ascending aorta area divided by the body height) to define a clinically-relevant aortopathy [10]. Bicuspid aortopathy (Figure 3B) is found in ca. 40–70% of BAV patients [11]. Aortopathy is less frequent in patients with a “normal” tricuspid aortic valve (so-called tricuspid aortopathy), which represents a wide spectrum of pathogenetically different diseases from congenitally-triggered Marfan syndrome in very young individuals to calcified atherosclerotic ascending aortic aneurysm in octogenarians [12].

### 1.3. Current Risk Stratification of Bicuspid Aortopathy

Bicuspid aortopathy was historically said to be associated with an increased life-long risk of severe aortic complications (aortic dissection and/or rupture) (Figure 3C) [13,14]. Recent data do not support this generalization [15], however indicates marked heterogeneity in the whole spectrum of BAV aortopathy and stresses the difficulties in predicting the individualized risk of aortic events [16].

Current risk stratification and treatment for bicuspid aortopathy is based on maximal aortic diameter, with greater diameter associated with a higher risk of an adverse event and exponentially-increasing risk with an ascending aorta diameter ≥60 mm or ≥4.25 cm/m^2^ [17,18]. The current guidelines for surgical treatment are based on a diameter of 55 mm in BAV patients without additional risk factors and 50 mm in those BAV patients with additional risk factors for dissection (e.g., family history of dissection, aortic growth rate ≥0.5 cm per year) [19].

### 1.4. Limitations of the Diameter-Based Risk Stratification

Most life-threatening aortic complications (especially dissection) cannot be prevented by following the current guidelines for surgical treatment, as recent data shows that >90% of aortic dissections occur with an aortic diameter that is less than 50–55 mm [20,21]. Therefore, the maximum aortic diameter alone cannot be used as a reliable parameter for risk stratification and the subsequent treatment of bicuspid aortopathy. Therefore, the clinical focus turned towards the identification of specific forms of bicuspid aortic valve disease (so-called BAV phenotypes) to improve the classification and risk prediction in BAV aortopathy.

### 1.5. Phenotype-Based Risk Stratification of Bicuspid Aortopathy

BAV is a highly heterogeneous disease. Previous studies identified two pathogenetically different BAV-associated valvulo-aortic phenotypes: (i) BAV stenosis with dilatation of the tubular ascending aorta and (ii) sinus of Valsalva dilatation with regurgitation of the BAV (root phenotype) [22,23,24]. There is some evidence that the severity of bicuspid aortopathy correlates with the respective phenotype of BAV [25]. In contrast to this finding, several prospective echocardiographic follow-up studies failed to show a significant association between BAV valve morphotype and the baseline aortic size as well as the progression rate of aortic dilatation [26,27]. Furthermore, longitudinal follow-up studies stressed the marked heterogeneity in the BAV aortopathy progression and the difficulty to discover fast progressors of aortopathy [27,28].

#### 1.5.1. The Phenotype of Bicuspid Aortic Stenosis with Dilatation of the Tubular Ascending Aorta

**Figure 4:**
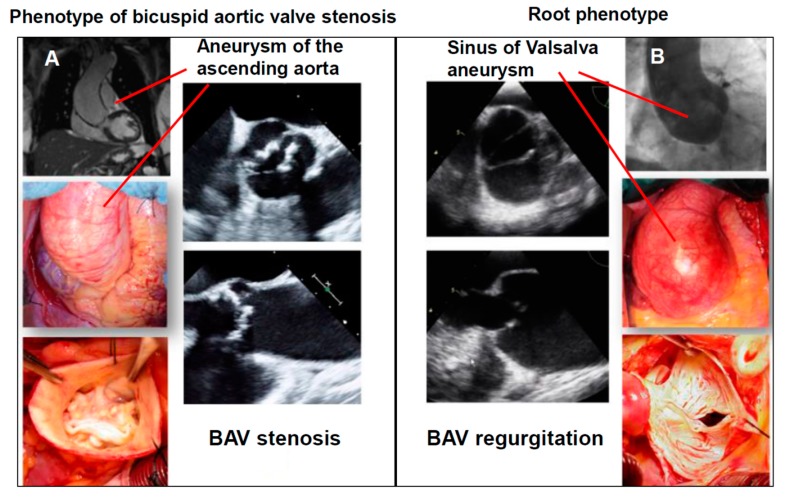
Clinical BAV Phenotypes. (**A**) BAV valvular stenosis with an asymmetric dilation of the tubular acending aorta; (**B**) root phenotype.

The most common clinical manifestation in BAV patients is the phenotype of BAV stenosis and the simultaneous asymmetrical dilation of the tubular ascending aorta. The common characteristics of this phenotype are a progressively calcific valvular stenosis, a mostly normal-sized sinus of the Valsalva segment, and asymmetrical dilatation of the tubular ascending aorta (Figure 4A). Most often, aortopathy is an incidental finding in BAV stenosis patients and remains in a stable condition over the long-term after an isolated surgical treatment of the aortic valve (i.e., without simultaneous aortic replacement) [22,29]. This aortic phenotype is supposed to be of predominantly hemodynamic genesis [30] that only very rarely shows a relevant progression after the isolated surgical correction of valvular stenosis.

#### 1.5.2. Root Phenotype (Dilatation of the Sinus of Valsalva in Combination with BAV Regurgitation)

The phenotype of a sinus of Valsalva dilatation including BAV regurgitation is known as a “root phenotype” (Figure 4B). Predominantly affected are male patients at a very young age. This phenotype of BAV bears many similarities to other congenital connective tissue diseases (e.g., Marfan syndrome). In accordance with the Marfan syndrome, this type of aortopathy might be associated with a “malign” genetically-triggered aortopathy. A recent echocardiographic follow-up study confirmed the increased rate of adverse aortic events in patients presenting with the root phenotype [23]. Furthermore, preliminary genetic analysis showed a high prevalence of rare deleterious mutations in BAV patients with a root phenotype [31]. In accordance with these findings, a previous meta-analysis demonstrated a 10-fold increased risk of post-AVR aortic dissection due to the root phenotype, compared with the phenotype of BAV valvular stenosis [32].

### 1.6. Current Knowledge on Genetics in BAV

Although only a single prospective study has specifically addressed the deleterious genetic variants in BAV root phenotype patients, several recent studies analyzed the impact of genetic abnormalities in the whole BAV population. Novel deleterious loss-of-function mutations were identified in GATA4, GATA6, and NKX2.5 genes and all showed an association with enhanced susceptibility to a BAV, thus providing insight into the molecular mechanisms underlying the BAV disease [33,34,35]. Interestingly, all of the identified mutations did not correlate with the specific BAV aortic phenotype, thus questioning their impact on the aortopathy. Therefore, the role of such genetic abnormalities in the development and progression of BAV-associated aortopathy still remains to be clarified.

### 1.7. Limitations of the Phenotype-Based Risk Stratification

**Figure 5:**
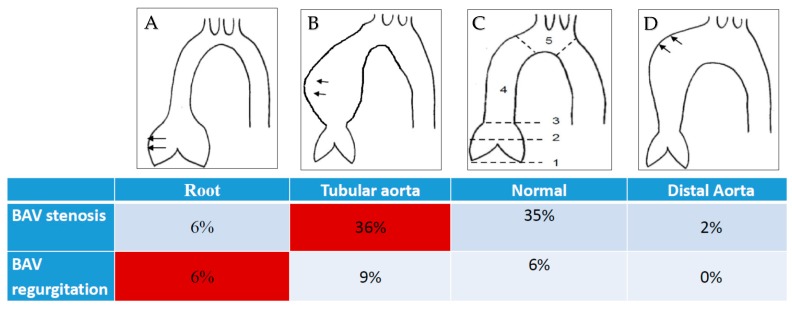
Forms of bicuspid aortopathy: (**A**) root phenotype; (**B**) dilation of ascending aorta; (**C**) normal aorta; (**D**) dilation of distal ascending aorta (proximal aortic arch). (1) aortic annulus; (2) sinus if Valsalva; (3) sinotubular junction; (4) tubular ascending aorta; (5) aortic arch. Only 42% of bicuspid patients are classified according to the two phenotypes (marker in red). The remaining 58% are not considered.

Although BAV-associated phenotypes differ significantly in terms of their clinical presentation and aortopathy risk, some major limitations of the phenotype-based risk stratification exist. Given the variability of valvular and aortic forms which are encountered in the BAV patients, there is a myriad of potential combinations of valvulo-aortic phenotypes available [36]. Individualized risk stratification in 80 different BAV valvulo-aortic phenotypes is only mechanistic and therefore, is not reasonable. Consecutive MRI analyses of a surgical BAV cohort showed that the two previously described BAV phenotypes (i.e., BAV stenosis and dilatation of the tubular ascending aorta and root phenotype) only account for 42% of the entire BAV cohort and are therefore insufficient to entirely depict the spectrum of this disorder (Figure 5A–D) [37].

Thus, the identification of additional diagnostic tools that can better predict risk for an individual BAV patient are urgently needed.

## 2. How to Overcome the Current Shortcomings in the Risk Stratification of BAV Aortopathy

Considering the above-mentioned limitations of diameter-based and phenotype-based risk assessment in BAV aortopathy, novel approaches are needed to create the basis for improved prognostic aortopathy risk stratification models. Prospective databases comprising clinical, biomarker, and longitudinal follow-up data are urgently needed to overcome the limitations of current aortopathy risk assessment. First, population-based epidemiology cohorts are needed to gain additional information about BAV distribution and aortic complications in the population. Furthermore, disease cohorts are required to prospectively investigate novel imaging techniques and circulating biomarkers.

Within the last years, large-scale epidemiologic and cohort studies with extensive biobanks have been established, such as the clinical BAV cohorts and the population-based Hamburg City Health Study (www.hchs.de). In this context, a cluster of epidemiology, clinical parameters, and biomaterial of each participant is collected and is used for systematic BAV aortopathy research.

### 2.1. Example of the Disease Cohort—BAV

BAV disease cohorts include (i) echocardiographic; (ii) surgical; (iii) BAV root phenotype; and (iv) rheological analysis cohorts. (i) Echocardiographic cohort—summarizes BAV patients who are examined and monitored at UKE Hamburg in the interdisciplinary BAV outpatient clinic (cooperation between cardiac surgeons, cardiologists, radiologists, and human geneticists); (ii) Surgical cohort—includes all BAV patients that are referred to our heart center with an indication for aortic valve/proximal aorta surgery; (iii) Root phenotype cohort—summarizes BAV patients with a root phenotype, who underwent previous valvular and aortic surgery for BAV regurgitation and simultaneous aortic aneurysm; (iv) Rheological analysis cohort—included BAV patients who underwent aortic valve surgery due to BAV stenosis and had preoperative cardiac MRI analysis to quantify their transvalvular flow patterns. Intraoperative aortic tissue samples were collected according to the MRI-based transvalvular flow analysis (i.e., aortic samples from the “jet-site” and “control-site”).

### 2.2. Population-Based Cohorts—Example Hamburg City Health Study (HCHS)

The HCHS is a population-based observational study of the population of Hamburg, which has examined the inhabitants of Hamburg between 45 and 75 years of age since February 2016. Between 2016 and 2021, a total of 45,000 participants will be included and examined in a standardized way according to standard operating procedures (SOPs). There is a comprehensive, 6-h standard examination including echocardiography during the first visit. BAV individuals are identified by transthoracic echocardiography which is approved by two independent echocardiography experts. Furthermore, blood samples are taken and are processed and stored in a large-scale biobank. To identify the probands with an increased risk of adverse cardiovascular events, risk scores are calculated. Medium to high risk probands undergo MRI examination of the heart and the thoracic aorta. Follow-up examinations are then conducted via questionnaires in regular 1-year intervals and are continued for at least 6 years. Re-examination at the HCHS center using the same standardized protocol are scheduled for 6 years after the initial visit. 

The combination of population and disease cohorts in aortopathy risk prediction has the major advantage of providing representative cohorts for integrated screening, validation, and prognostic value examinations of potential biomarkers which are only possible in a longitudinal, large-scale population-based epidemiological study.

## 3. BAV and Circulating Biomarkers for the Risk Stratification in Bicuspid Aortopathy: Current Knowledge

There is a growing interest to use circulating biomarkers for risk stratification in patients with aortopathy. The routine use of such biomarkers in clinical practice is currently limited due to the lack of their validation in the multicenter prospective trials and an unclear association between the expression of such biomarkers and clinically-relevant cardiovascular events. So far, investigations have focused on the following biomarkers: matrix metalloproteinases (MMPs), tissue inhibitors of matrix metalloproteinases (TIMPs), transforming growth factor ß (TGF-ß), alpha1-antitrypsin, soluble receptor for advanced glycation end products (sRAGE), as well as non-coding microRNAs [38,39,40,41,42,43]. All of these biomarkers can be conceptually subdivided into protein-based biomarkers (i.e., MMPs, TIMPs, TGF-ß, alpha1-antitrypsin, sRAGE) and non-coding RNAs.

### 3.1. Protein-Based Biomarkers

Matrix metalloproteinases (MMPs) and their tissue inhibitors (TIMPs) are the key players in the regulation of extracellular matrix (ECM) remodeling [39] and thereby have a major impact on the maintenance of the blood vessel walls’ homeostasis. MMP-2 and MMP-9 were found to be highly expressed in BAV patients compared with their tricuspid counterparts and MMP-2 showed a strong linear correlation with the proximal aortic diameter [44]. TIMP-1, TIMP-2, TIMP-3, and TIMP-4 were similarly found to be significantly increased in the aortic tissue of BAV patients, although without a significant correlation with the proximal aortic diameters [45]. Transforming growth factor-ß (TGF-ß) is a soluble cytokine that has an impact on vascular remodeling processes and has been shown to be increased in BAV patients as well as in those with congenital aortopathies [40]. Alpha1-antitrypsin is the best-known circulating protease inhibitor that controls the activity of proteolytic enzymes in the human tissue including the blood vessel wall. Locally reduced alpha1-antitrypsin levels have been demonstrated in the wall of acutely dissected ascending aorta as compared to healthy aortic tissue [46]. The soluble receptor for the advanced glycation end-product (sRAGE) is a circulating ligand member of the immunoglobulin superfamily that mediates immunogenic and proinflammatory signals from so-called RAGE ligands and thereby, the progression of vasculopathy [47].

### 3.2. Non-Coding RNAs

Non-coding RNAs (ncRNA) are functional RNA molecules that are transcribed from DNA but are not translated into proteins. NcRNAs have an important function to regulate gene expression at the transcriptional and post-transcriptional level. Those ncRNAs that are involved in the regulation of the information flow from DNA to protein can be subdivided into transacting microRNAs (miRNAs), long non-coding RNAs (lncRNAs), and small nuclear RNAs (snRNA).

MicroRNAs (miRNAs) are coded by their own genes as pre-miRNAs and exert a very wide spectrum of regulatory activities at the molecular and cellular level by binding to a specific target messenger RNA with a complementary sequence to induce cleavage, degradation, or block translation. Recent studies led to the assumption that specific miRNAs, through the regulation of the MMPs/TIMPs homeostasis, are involved in cardiac matrix remodeling [42], a process that is also pathogenetically comparable to the dilation of the proximal aorta. Circulating miRNAs are supposed to be directly linked to the development and progression of aortic diseases [48].

**Figure 6:**
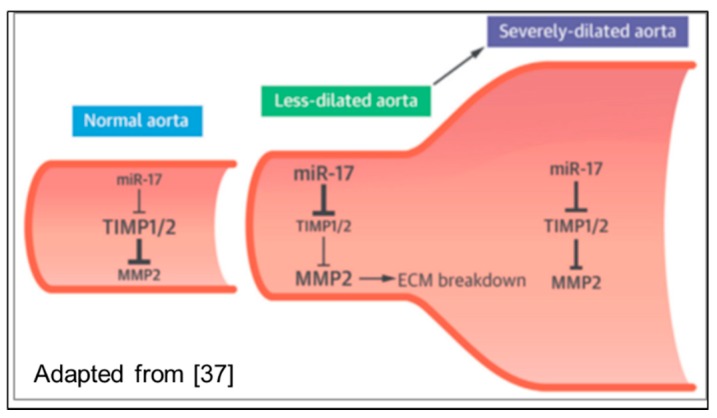
Impact of miR-17 on TIMP/MMP homeostasis through regulation of TIMP expression. Adapted from [50].

This hypothesis is supported by a recently published study [49], showing that dilation of the ascending aorta progresses through miR-17 over-expression at an early stage, leading to a decreasing TIMP1/2 activity and the subsequent activation of MMP2 (Figure 6).

In addition, further studies postulate the potential influence of other circulating miRNAs: miR-21, miR-26, miR-29, miR-122, miR-130a, miR-133a, and miR-143/145 on the pathogenesis of proximal aortic aneurysms and acute aortic syndrome [50,51]. 

A recent study by Martinex-Micaelo et al. [50] applied a miRNome-wide microarray approach to identify the circulating miRNAs that are specifically associated with BAV and aortic dilation. In addition to the microarray analysis covering 1205 human miRNAs in 24 individuals (i.e., 18 BAV and 6 TAV patients), the plasma microRNA expression was validated by real-time qRT-PCR in an independent patient cohort. This validation cohort included 43 individuals followed by echocardiography at a single institution with (a) BAV and no aortic dilatation (*n* = 12); (b) BAV with aortic dilatation (*n* = 12); (c) TAV without aortic dilatation (*n* = 12); and (d) TAV with aortic dilatation (*n* = 7). The authors revealed that the expression levels of the circulating miR-122, miR-130a, and miR-486 correlated significantly with the morphology of the aortic valve (i.e., BAV vs. TAV), while the expression pattern of miR-718 in the plasma was strongly influenced by the dilation of the ascending aorta.

Another recent publication by Borghini et al. [52] focused on the miRNAs expression in the aneurysmal aortic tissue. The authors employed next generation sequencing to analyze the entire miRNome expression in the aortic tissue of 7 BAV patients vs. 6 TAV patients with aortopathy. A total of 12 miRNAs were found to be differentially expressed in BAV aortic samples as compared to TAV samples. The functional pathway analysis revealed different pathways that were linked to BAV and aneurysm formation, including the Hippo signaling pathway, ErbB signaling, TGF-β signaling, and focal adhesion. The subsequent validation analysis of the 12 identified miRNAs in the cohort of 60 patients (i.e., 30 BAV aortic samples and 30 TAV samples) confirmed the significant downregulation of miR-424-3p (*p* = 0.01) and miR-3688-3p (*p* = 0.03) in BAV patients as compared to their TAV counterparts. Two major limitations of this otherwise elegant analysis are (1) the lack of data regarding the expression patterns of the circulating miRNAs and (2) the presence of significant phenotypic variability between the analyzed BAV and TAV cohorts (i.e., the majority of the included BAV patients had valvular stenosis with supracoronary ascending aortic aneurysm, while valvular regurgitation with presumably root dilatation was the predominant valvular lesion in the TAV cohort).

The impact of circulating and aortic tissue miRNAs to identify the progression of BAV-associated aortopathy has been highlighted in a very recent review by Maredia and co-authors [52]. These authors stressed the importance of a multimarker approach in combination with the modern clinical imaging techniques to predict the aortopathy progression in BAV patients. Furthermore, the potential role of epigenetic biomarkers, such as DNA methylation and histone modifications which may interact in the development of BAV-associated aortopathy have been addressed [52].

**Figure 7:**
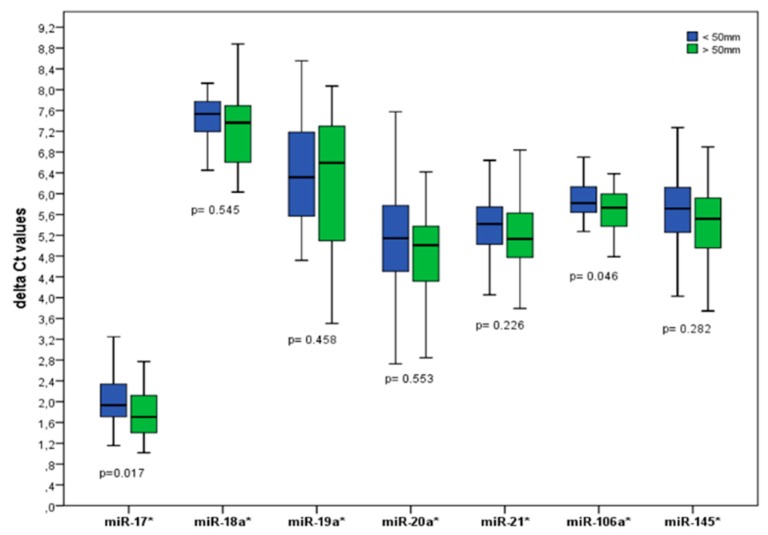
Circulating microRNAs in severely dilated aorta >50 mm (SD subgroup) vs. less dilated bicuspid aorta (LD subgroup) <50 mm. Delta Ct values normalized to miR-16 are shown. *p*-Values shown for the comparison of SD vs. LD subgroup.

In a surgical cohort of BAV patients with aortopathy, an expression analysis of 7 circulating microRNAs (miR-17, miR-18a, miR-19a, miR-20a, miR-21, miR-106a, miR-145) showed differential levels of these microRNAs in patients with a severely-dilated (i.e., >50 mm diameter) vs. less-dilated (<50 mm diameter) proximal aorta (Figure 7). These results are in accordance with the findings by Wu et al. [48], who showed increased miR-17/miR-106a expression in an early stage of progressive aortic dilation.

**Figure 8:**
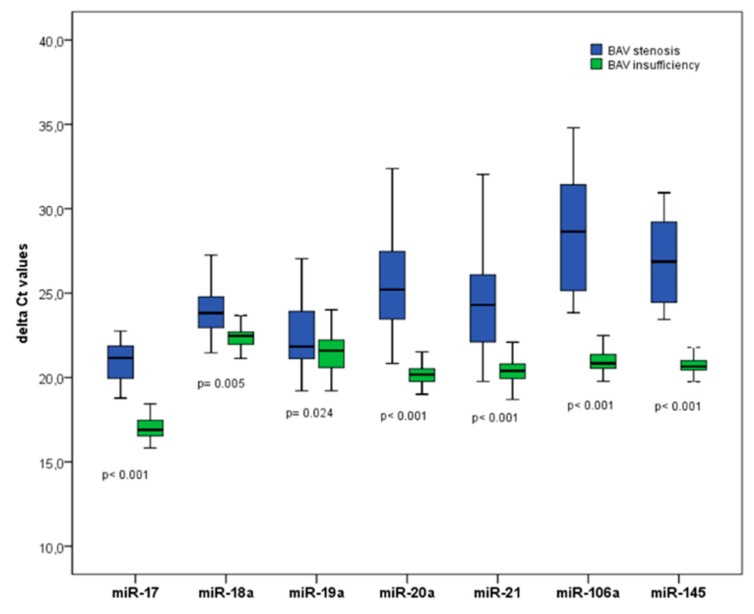
Circulating microRNAs in a patient cohort with BAV regurgitation (BAV-AR) vs. BAV stenosis (BAV-AS). Delta Ct values are shown. *p*-Values shown for the comparison of BAV stenosis vs. BAV insufficiency subgroup.

Furthermore, the expression of the 7 above mentioned microRNAs (miR-17, miR-18a, miR-19a, miR-20a, miR-21, miR-106a, miR-145) was significantly different in a BAV valvular stenosis (BAV-AS) vs. a BAV regurgitation (BAV-AR) population (Figure 8). All 7 investigated microRNAs were significantly downregulated in a subgroup of patients with BAV regurgitation (BAV-AR). BAV patients in the BAV-AR cohort had a significantly more severe bicuspid aortopathy, as the maximum aortic diameter was significantly increased (48 ± 9 mm in the BAV-AR cohort vs. 39 ± 12 mm in the BAV-AS cohort, *p* = 0.001).

A correlation analysis of the maximum aortic diameter in the patients with the bicuspid aortopathy and the expression of the investigated microRNAs revealed a significant inverse linear correlation with the maximum aortic diameter, particularly for circulating miR-17/miR-20a [53].

## 4. Summary

BAV disease and the associated aortopathy represent a heterogeneous disorder with diverse clinical manifestations. The individual risk stratification of adverse cardiovascular events that is based on the maximum aortic diameter and two clinical phenotypes (dilation of ascending aorta and root phenotype) is still insufficient. Circulating biomarkers and especially microRNAs are offering promising preliminary results to improve the prognostic risk stratification and individualized treatment of bicuspid aortopathy.

Efforts in the following years will focus on the establishment of circulating microRNAs as biomarkers of bicuspid aortopathy. Comprehensive analysis of disease cohorts in combination with the large-scale prospective, population-based studies will be able to elucidate the causal relationship between circulating miRNAs and the prognosis of bicuspid aortopathy. The proof of such a relationship may serve as a basis for further follow-up and therapeutic strategies and aortopathy treatment algorithms. The identification of additional (circulating) non-coding RNAs that are associated with bicuspid aortopathy as prognostically relevant biomarkers would have a major impact on the development of personalized cardiovascular medicine and the long-term follow-up of BAV patients.
